# Using social autopsy to understand maternal, newborn, and child mortality in low-resource settings: a systematic review of the literature

**DOI:** 10.1080/16549716.2017.1413917

**Published:** 2017-12-20

**Authors:** Cheryl A. Moyer, Cassidy Johnson, Elizabeth Kaselitz, Raymond Aborigo

**Affiliations:** ^a^ Departments of Learning Health Sciences and Obstetrics & Gynecology, University of Michigan Medical School, Ann Arbor, MI, USA; ^b^ Global REACH, University of Michigan Medical School, Ann Arbor, MI, USA; ^c^ Navrongo Health Research Centre, Navrongo, UE/R Ghana

**Keywords:** Social autopsy, verbal autopsy, maternal mortality, neonatal mortality, infant mortality, developing countries

## Abstract

**Background**: Social, cultural, and behavioral factors are often potent upstream contributors to maternal, neonatal, and child mortality, especially in low- and middle-income countries (LMICs). Social autopsy is one method of identifying the impact of such factors, yet it is unclear how social autopsy methods are being used in LMICs.

**Objective:** This study aimed to identify the most common social autopsy instruments, describe overarching findings across populations and geography, and identify gaps in the existing social autopsy literature.

**Methods**: A systematic search of the peer-reviewed literature from 2005 to 2016 was conducted. Studies were included if they were conducted in an LMIC, focused on maternal/neonatal/infant/child health, reported on the results of original research, and explicitly mentioned the use of a social autopsy tool.

**Results**: Sixteen articles out of 1950 citations were included, representing research conducted in 11 countries. Five different tools were described, with two primary conceptual frameworks used to guide analysis: Pathway to Survival and Three Delays models. Studies varied in methods for identifying deaths, and recall periods for respondents ranged from 6 weeks to 5+ years. Across studies, recognition of danger signs appeared to be high, while subsequent care-seeking was inconsistent. Cost, distance to facility, and transportation issues were frequently cited barriers to care-seeking, however, additional barriers were reported that varied by location. Gaps in the social autopsy literature include the lack of: harmonized tools and analytical methods that allow for cross-study comparisons, discussion of complexity of decision making for care seeking, qualitative narratives that address inconsistencies in responses, and the explicit inclusion of perspectives from husbands and fathers.

**Conclusion**: Despite the nascence of the field, research across 11 countries has included social autopsy methods, using a variety of tools, sampling methods, and analytical frameworks to determine how social factors impact maternal, neonatal, and child health outcomes.

## Background

Social, cultural, and behavioral factors are often potent upstream contributors to poor maternal, neonatal, and child health outcomes, especially in low- and middle-income countries (LMICs). While anatomical autopsies and verbal autopsies (typically interviews with family members about the days leading up to death) identify leading clinical causes of death, social autopsy aims to push beyond the medical causes of mortality. Social autopsy – which includes verbal autopsy questions and a host of additional sociocultural and behavioral questions – seeks to discern the social, behavioral, and health system factors that contribute to deaths [1,2]. Such factors could include access and transportation to medical care, care-seeking behaviors, cultural norms surrounding illness, and local health system practices – which may not be evident when looking at biomedical causes alone.

**Figure 1.**
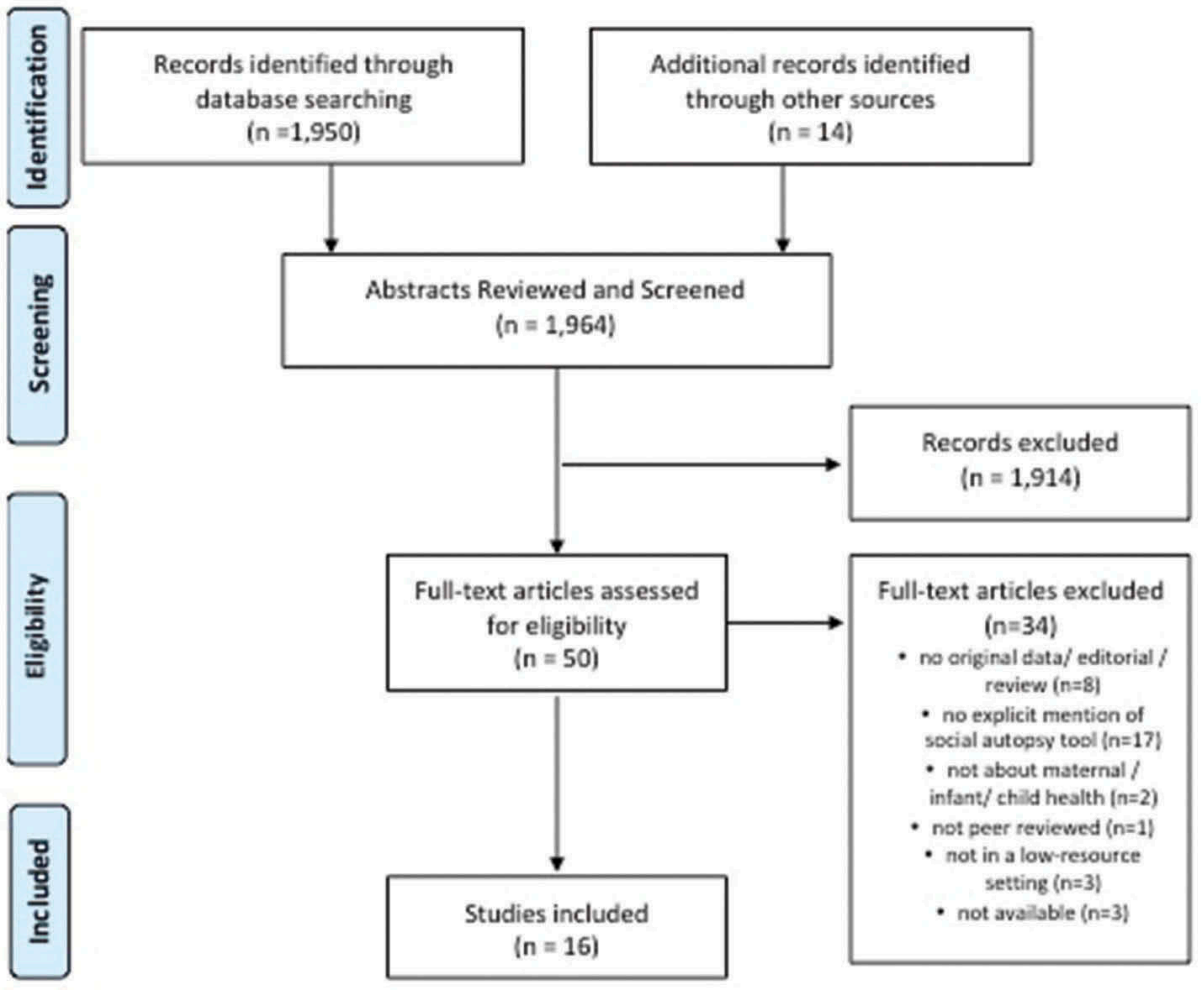
PRISMA flow diagram (adapted from Moher et al. 2009).

Adding social autopsy questions to verbal autopsy tools can create a more robust understanding of the modifiable factors leading to deaths and allow for more expertly tailored interventions [1]. For example, a verbal autopsy might attribute the death of a newborn to neonatal sepsis. While this is valuable information, it would not be clear whether the sepsis was likely a result of maternal sepsis, a non-sterile delivery in a health facility, a non-sterile home delivery, or some other cause. Adding a social autopsy component might reveal that in some locations it is not uncommon to apply non-sterile substances to the umbilical cord, such as shea-nut butter or cow dung, and it is often the grandmothers who insist upon such practices [3]. This information could change the focus of an intervention from facility-based efforts to community-level outreach to extended family members of new mothers.

This paper builds on the 2011 comprehensive review of Kalter et al. [2] to provide an update of the peer-reviewed social autopsy literature and: (1) determine the extent to which Social Autopsy methodology has been used in low- and middle-income countries to understand the contributors to maternal, newborn and child mortality; (2) identify the most common Social Autopsy instruments being used, as well as commonalities and differences in methodologies; (3) describe overarching findings across disparate populations and geographic areas; and (4) describe the quality of the evidence to date and identify potential gaps in the existing research literature.

## Methods

### Search strategy

A systematic search of the peer-reviewed, published literature from 2005 to 2016 was conducted to identify original research using social autopsy methodology. The year 2005 was chosen to ensure sufficient overlap with Kalter et al. (2011) comprehensive literature review [2], given that this systematic review methodology was designed to be more rigorous than a comprehensive review and include every article in the peer-reviewed literature that used specifically designated social autopsy methodology. We chose not to go farther back than 2005 because social autopsy methodology was the focus of this systematic review, and formalized social autopsy methods have only come into common usage within the past 10–15 years. Searches used: Ovid MEDLINE, International Pharmaceutical Abstracts, Journals@Ovid Full Text, Epub ahead of print, Global Health database, PubMed, Popline, and BioMed Central. Initial searches were conducted by two of the authors (CAM, CJ) on 10 August 2016, and repeated on 20 August 2016.

Given our explicit focus on research using ‘social autopsy’ methods (as opposed to the many researchers who explore the social determinants of health or social factors related to death), the following search terms were used in various combinations: social autopsy, psycho-social autopsy, social + autopsy, psychosocial + autopsy (search strategy available upon request). Additional hand searching was conducted by reviewing the references of all retrieved studies.

### Study selection and data extraction

Studies were included in the review if they were published in a peer-reviewed journal in English between January 2005 and August 2016, were conducted entirely or in part in a low- or middle-income country, focused on maternal/neonatal/infant/child health, reported on the results of original research, and explicitly mentioned the use of a social autopsy instrument, tool, survey, interview guide, or questionnaire. Review articles were not included, as this review aimed to focus on primary sources. Review articles were used to verify the identification of and inclusion of all articles that met the search criteria.

Study inclusion was determined in a multi-step procedure. First, two of the authors (CAM, CJ) evaluated bibliographic data and abstracts for concordance with formal inclusion rules. Studies that clearly did not meet the remaining inclusion criteria were discarded. The remaining studies were selected for full-text retrieval. Publications that did not present empirical data or meet inclusion criteria were discarded, but not before hand-searching the references. Full-text of studies identified from the references were retrieved as well. In a final step, two of the authors (CAM, CJ) examined the remaining studies in detail to identify the final sample of studies meeting all inclusion criteria.

### Assessment of quality

The USA National Institutes of Health ‘Quality Assessment Tool for Observational Cohort and Cross-Sectional Studies’ was adapted for use in this research [4]. This instrument includes 14 items that assess various components of a research study, from clear presentation of the research question through such factors as whether the assessors were blinded to the exposure status of participants. Eight of the 14 items were deemed to be appropriate for assessing the studies included in this review, although not every item was appropriate for every study (See Table 1.) Two authors (CAM, EK) separately reviewed each article against the quality checklist and then compared their individual ratings. In all but two cases, ratings were identical. For the two articles with disparate scores, the authors discussed their perspectives and reached consensus on a final score. A score between 0 and 1 was then calculated for each article, reflecting the number of ‘yes’ answers on the checklist relative to the number of items that were determined to be applicable to the study in question (e.g. 6 out of 7 possible yes answers yields a score of 0.86, whereas 7 out of a possible 8 yes answers yields a score of 0.88).

**Table 1.** Quality assessment tool, adapted from US national institutes of health [5].

	Yes	No*	Other**
1. Was the research question or objective in this paper clearly stated?	1	0	–
2. Was the study population clearly specified and defined?	1	0	–
3. Was the participation rate of eligible persons at least 50%	1	0	–
4. Were all the subjects selected or recruited from the same or similar populations (including the same time period)? Were inclusion and exclusion criteria for being in the study pre-specified and applied uniformly to all participants?	1	0	–
5. Was a sample size justification, power description, or variance and effect estimates provided?	1	0	–
6. For determinants that can vary in amount or level, did the study examine different levels of the determinant as related to the outcome? (e.g. categories of determinant, or determinant measured as a continuous variable)	1	0	–
7. Were the determinant measures (independent variables) clearly defined, valid, reliable, and implemented consistently across all study participants?	1	0	–
8. Were the outcome measures (dependent variables) clearly defined, valid, reliable, and implemented consistently across all study participants?	1	0	–

*Includes not reported and cannot determine.

**Not applicable.

### Analysis and synthesis strategy

Given the variety and types of studies included in this systematic review – including descriptive and evaluative studies that ranged from simple bivariate analyses to complex multivariate modeling – a meta-analysis was neither possible nor appropriate.

One table was created that synthesized the findings across all selected studies, including the study design, the countries in which the research was conducted, methods for identifying respondents, the number and types of respondents interviewed, the recall period, the study’s main findings, the social autopsy instrument used, and the conceptual model used to drive the analysis. Another table was used to juxtapose quantitative findings regarding care-seeking across studies and by neonatal, child, and maternal events. These tables were used to address the main research questions.

## Results

Figure 1 (adapted from Moher et al. [4]) illustrates the process of identifying, screening, and selecting the articles included in this review. A total of 1,950 citations were identified through a systematic protocol of electronic searches, with an additional 14 records identified through other sources. After removing those articles that clearly did not meet the inclusion criteria upon review of the abstracts, a total of 50 articles were identified for full text review. At this final stage another 34 were removed for such reasons as not including original data (n = 8), not mentioning or citing a social autopsy tool (n = 17), not addressing maternal/infant/child health (n = 2), not being peer reviewed (n = 1), not occurring in LMIC (n = 3), or not being available (n = 3). This left 16 published studies that met all inclusion criteria and for which data were extracted (see Table 1).

To address the first objective of this review (to determine the extent to which Social Autopsy methodology has been used in low- and middle-income countries to understand the contributors to maternal, newborn, and child mortality), Table 2 illustrates that the 16 articles included in this systematic review represent social autopsy research conducted in 11 different countries. India, Niger, and Uganda were the sites of several research studies, whereas Bangladesh, Cameroon, Ghana, Indonesia, Kenya, Malawi, Senegal, and South Africa were represented by single studies. Most of the studies included a focus on the neonatal period (N = 8), with six studies including a focus on children under age 5, five studies focusing on maternal mortality, and three studies focusing on infants under the age of 1.

**Table 2.** Selected articles that met inclusion criteria.

Author	Year	Country	Study Design	Sampling	Respondents	Target Age	Recall Period	Main Factor/Finding	Instruments Used	Conceptual Model used to Drive Analysis	Quality Score
D’Ambruoso L, Byass P, Qomariyah SN. [6]	2010	Indonesia	Cross-sectional	Village-based informants, volunteer health workers, and unpaid village officials collect info via survey of maternal deaths from Jan. 2004 to Dec. 2005.	Caregivers (N not reported) interviewed about 104 mothers	Mothers	Not reported	Nearly half reported delay in decision to seek care (42%) 2/3 reported delays in seeking care (66%) Almost half reported delays in receiving care (44%)	WHO Verbal Autopsy Tool; Undefined Social Autopsy Tool	Three Delays Model	1.0
Deshmukh V, Lahariya C, Krishnamurthy S, Das MK, Pandey RM, Arora NK. [7]	2016	India	Cross-sectional	National Family Health Survey – 2 data to detect under 5, infant, and neonatal mortality rate between Apr. 2005 and Mar. 2006.	Caregivers (N not reported) surveyed about 1,488 under 5, infant, and neonate deaths.	Under 5 years, neonates, and infants	Not reported	52.6% of neonates, 24.4% of infants, and 19.4% of children were not taken to a health facility before death. Delay at home occurred in 62.5% of cases of neonates. Out of 296 newborns taken to health facility, 27.7% reported difficulties in transit. For post-neonatal deaths, delay at home occurred in 42.3%. In more than half (58.7%) of these, delay was due to waiting for home remedy to take effect. Delay and difficulties in transit (46%), were primarily due to transport problems and lack of social support for carrying the sick child.	International Clinical Epidemiology Network (INCLEN) Verbal Autopsy instrument	Three Delays Model	1.0
Hildenwall H, Tomson G, Kaija J, Pariyo G, Peterson S. [8]	2008	Uganda	Cross-sectional	Iganga/Mayuga Demographic Surveillance Site used to identify under 5 deaths between Mar. and June 2006.	Caregivers (N = 26) interviewed about 26 child deaths	Under 5 years, neonates, and infants	4–6 weeks	All but 3 children had been taken to an allopathic healthcare provider during the illness, while the other 3 had been taken to a traditional care provider. 3 mothers reported having to find the father before any decision could be made. (21/26) were taken to more than one healthcare provider. Barriers to care related to 1) Illness interpretation, 2) seeking care, 3) receiving adequate treatment.	INDEPTH Network Verbal tool; Aguilar et al.’s Social Autopsy Tool from Bolivia	Not clear	0.67
Jat TR, Deo PR, Goicolea I, Hurtig A, San Sabastian M. [9]	2015	India	Cross-sectional	Using ‘government records and community informants’ purposively selected maternal deaths to cover range of circumstances July 2011-Nov. 2011.	31 caregivers interviewed about 22 maternal deaths.	Mothers	2 weeks – 1 year	21/22 women were delayed in reaching care. 13/22 women accessed more than 2 health facilities. 13/22 women were delayed at health facility from initiation of care. 12 died at facility; 9 died en route	WHO Verbal Autopsy Tool; CHERG Social Autopsy Tool	Three Delays Model	0.80
Källander K, Hildenwall H, Waiswa P, Galiwango E, Peterson S, Pariyo G. [10]	2008	Uganda	Cross-sectional	Iganga/Mayuga Demographic Surveillance Site used to identify under 5 childhood deaths occurring between Nov. 2005 and Aug. 2007	Caregivers (N not reported) surveyed about 164 children.	Under 5 years old	4–6 weeks	Study collected info on many diseases, but emphasizes results for pneumonia Overall, a child that died of pneumonia had been sick for 7 days Median reported duration was 4 hours from illness recognition to home care initiation, 2 days until care sought outside the home 60% were within one hour walking distance of the facility visited. 14% never taken outside of the home. Delayed care seeking was only associated with home treatment with antibiotics.	INDEPTH Network Verbal tool; Kalter et al.’s Social Autopsy Tool from Bolivia	Not clear	0.71
Källander K, Kadobera D, Williams TN, Nielsen RT, Yevoo L, Mutebi A, Akpakli J, Narh C, Gyapong M, Amu A, Waiswa P. [11]	2011	Uganda/Ghana	Cross-sectional	Iganga/Mayuga Demographic Surveillance Site used to identify child deaths from Jan. 2009-July 2010 and Dodowa HDSS from Dec. 2008 -Dec. 2009.	Caregivers (N = 474) were interviewed about 474 child deaths.	Under 5 years old	4–6 weeks	96% in Iganga/Mayuge and 70% in Dodowa recognized severe symptom prior to death; 32 and 80% were first treated at home. Half of caretakers in Iganga/Mayuge adhered to provider’s referral advice. Of those who did not, 87% cited cost. 44% of delays for Iganga/Mayuge were caused by problems at the facility. 33% cited problems with transport, with 24% reported living 2+ hours to facility. In Dodowa, 63% of delays were caused by factors in the household. 82% of caretakers who recognized a severe symptom still waited more than a day before they went for treatment.	INDEPTH Network Verbal and Social Autopsy Tool	Pathway to Survival; Three Delays Model	1.0
Kalter HD, Yaroh AG, Maina A, Koffi AK, Bensaïd K, Amouzou A, Black RE. [12]	2016	Niger	Cross-sectional	Deaths identified by the 2010 Niger National Mortality Survey, with deaths as far back as 2006.	453 caregivers interviewed about 453 neonatal deaths. Some secondary respondents to fill gaps.	Neonatal	Data collection spanned from March-September 2012 for deaths dating back to 2006.	Women with pregnancy complications were no more likely than mothers without complications to deliver at a health facility (32.7% vs. 25.8%). Although 95.8% of caregivers reported there was a serious or severe symptom, 60.3% received no care for their illness. Of the 90 who did not seek care, 28.7% reported 2 constraints on average, including cost of transportation or health care, lack of transportation, and distance to facility	Population Health Metrics Research Consortium Verbal Autopsy Questionnaire; CHERG Social Autopsy Questionnaire	Pathway to Survival	1.0
Koffi AK, Maina A, Yaroh AG, Habi O, Bensaïd K, Kalter HD. [13]	2016	Niger	Cross-sectional	Deaths identified by the Niger National Mortality Survey conducted July-Aug. 2010.	Caregivers (N = 550) interviewed about 601 children	Under 5 years old	2–5 years with a mean of 2.7 years	Of the 601 children tracked through the Pathway to Survival model, it would take 62.4% about 67 minutes to reach the first health care provider. About 113 of the caregivers stated that distance, lack of transport, and cost were the biggest constraints to seeking care at a health facility. 96.2% of caregivers recognized that their child had a severe symptom. Median length from illness onset to care-seeking was 1 day.	Population Health Metrics Research Consortium Verbal Autopsy Questionnaire; CHERG Social Autopsy Questionnaire	Pathway to Survival	1.0
Koffi AK, Mleme T, Nsona H, Banda B, Amouzou A, Kalter HD. [14]	2015	Malawi	Cross-sectional	Deaths identified by a 24,000 household survey for the Real-time Mortality Monitoring Project from Oct. 2011-Feb. 2012	320 caregivers interviewed about 320 neonatal deaths.	Neonatal	4 years	Of the 180 of the newborns born at home or leaving health facility alive, 97.8% of caretakers recognized a severe or possibly severe symptom. Only 61.1% of the caregivers sought or tried to seek care. 79% of them first sought care outside of the home. Median length of delay from onset to seeking formal care was 1 day. 81.3% of newborns for whom formal care was sought reached provider after an average of 91 minutes. Among constraints, most important were distance (52–74%), lack of transport(49–68%), and cost (21–74%).	Population Health Metrics Research Consortium Verbal Autopsy Questionnaire; CHERG Social Autopsy Questionnaire	Pathway to Survival	1.0
Koffi AK, Libite PR, Moluh S, Wounang R, Kalter HD. [15]	2015	Eastern Region of Cameroon	Cross-sectional	Deaths identified by a census of 16,954 households from Oct.-Dec. 2010 (all child deaths in last 10 years)	164 mothers interviewed about 164 neonatal deaths.	Under 5 years old	4 years	Of the 123 neonates who did not die at the facility where delivered, 98% of caregivers recognized severe or possibly severe symptom, yet only 44.7% sought or tried to seek care. Of 77 mothers who delivered outside a health facility and faced multiple constraints, 73% reported cost as main barrier, with approximately a third reporting too far to travel and lack of transportation.	Population Health Metrics Research Consortium Verbal Autopsy Questionnaire; CHERG Social Autopsy Questionnaire	Pathway to Survival	1.0
Moshabela M, Sene M, Nanne I, Tankoano Y, Schaefer J, Niang O, Sachs SE. [16]	2015	Senegal	Cross-sectional/Case-series	Deaths identified by active household surveys, using mHealth platform Childcare+.	Caregivers (N not reported) interviewed about 5 maternal deaths	Maternal	1–2 weeks	4 of the 5 case reports describe death due to post-partum hemorrhage 3 of the 5 had emergency cesarean section deliveries 2 of the 5 had seen traditional providers and/or used traditional medicines	MVP VASA Tool, which combines the WHO Verbal Autopsy tool with an expanded section of social contributors	Pathway to Survival	0.88
Njuki R, Kimani J, Obare F, Warren C. [17]	2014	Kenya	Cross-sectional	Death audits for HIV-related deaths among women living within 5 km of specific facilities in 3 districts (deaths 1996–2010).	Caregivers (N not reported) about 218 HIV/AIDS-related deaths among women aged 15–49	Women of Reproductive Age	Data collection in 2010 for deaths as far back as 1996	Delays associated with: poor knowledge and understanding of AIDS-related illness, distance to facility, transportation costs, medical pluralism, stigma, low HIV perception, lack of family support and health care system barriers.	WHO Verbal Autopsy Questionnaire, Social Autopsy Tool unclear	Not clear	1.0
Nonyane BAS, Kasmi N, Koffi AK, Begum N, Ahmed S, Baqui AH, Kalter HD. [18]	2016	Bangla-desh	Cross-sectional	Newborn deaths identified in four sub-districts between Oct. 2007 and May 2011.	Caregivers (N = 319) interviewed about 331 deaths.	Neonatal	2.5 years	Of 165 mothers reporting concerns/barriers, 60% reported the most common barrier as cost. 18% said they thought the baby would die anyway. 16% thought the baby needed traditional care. 12% cited too late at night to travel and 11% cited distance to formal care facility.	WHO Verbal Autopsy Questionnaire, CHERG Social Autopsy Tool	Not clear	1.0
Tlebere P, Jackson D, Loveday M, Matizirofa L, Mbombo N, Doherty T, Wigton A, Treger L, Chopra M. [19]	2007	South Africa	Cross-sectional	Mothers (HIV±) who participated in previous PMCTC study; mothers from same communities; community health workers Identified any woman who had delivered a baby in the last 9–12 months	Infant deaths sampled from PMTCT cohort study (N = 75); maternal deaths identified from PMTCT study as well as local hospital registry and community health workers (N = 18); Families interviewed at their homes.	Maternal deaths; newborn deaths	Not reported	Most maternal deaths were related to HIV/AIDS (67%). Most women sought care without delay at the beginning of their illness. Mean age of infants at death was 16.7 weeks, infectious causes predominant. Themes identified as constraints in service utilization included: lack of money for transportation to the facility, attributing the cause of the illness to witchcraft, lack of awareness of danger signs, poor quality of care, and the role of traditional healers.	WHO Verbal Autopsy Questionnaire, Social Autopsy Tool unclear	Not clear	1.0
Upadhyay RP, Rai SK, Krishnan A. [20]	2013	India	Cross-sectional	28 villages of Comprehensive Rural Health Services Project, All India Institute of Medical Sciences; neonatal deaths in 2010.	Caregivers (N not reported) interviewed about 50 neonatal deaths.	Neonatal	Not reported	Delay in deciding to seek care identified in 44% of cases. Transportation delay reported in 35% of cases. Household and transport related delays were top contributors to deaths in newborns.	INDEPTH Network Verbal and Social Autopsy Tool	Three Delays Model	1.0
Waiswa P, Kallander K, Peterson S, Tomson G, Pariyo GW. [21]	2010	Uganda	Cross-sectional	Makerere University/Mayuge Health and Demographic Surveillance Site; deaths from Jan. 2005-Dec. 2008.	Caregivers (N not reported) surveyed about 64 neonatal deaths.	Neonatal	4–6 Weeks	Delays in problem recognition or deciding to seek care cited in 50% of cases. Delay in receiving quality care at health facility reported in 30% of cases. Transport delay identified in 20% of cases. Median time to seek care was 3 days. Health facilities did not have adequate capacity for newborn care and health workers did not have adequate knowledge of newborn care assessed via survey.	INDEPTH Network Verbal Autopsy tool and CHERG Social Autopsy tool.	Three Delays Model	0.86

To address the second objective of this review (to identify the most common Social Autopsy instruments being used, as well as commonalities and differences in methodologies). Table 2 illustrates that the instruments used to conduct social autopsies varied across the 16 published manuscripts, and Table 3 describes the instruments in more detail based upon what was reported in the literature, which did not typically include the instruments themselves, detailed descriptions of the domains, or details on the structure or complexity of the tool. Five sources for the instruments were described, sometimes distinguishing between verbal autopsy and social autopsy, and other times combining the two. Six articles cited the Child Health Epidemiology Research Group’s social autopsy tool [22]; six cited the World Health Organization’s verbal autopsy tool [24]; five cited the INDEPTH Network’s verbal and social autopsy tool [23]; four cited the Population Health Metrics Research Consortium’s verbal autopsy tool [25]; and one cited the International Clinical Epidemiology Network (INCLEN) verbal autopsy tool [7]. Four of the 16 manuscripts did not describe the social autopsy tool in sufficient detail to be classified. Overall, the two most commonly used instruments for social autopsy assessment were the Child Health Epidemiology Research Group’s social autopsy tool and the INDEPTH network’s combined verbal and social autopsy tool.

**Table 3.** A comparison of social autopsy tools as reported in the literature.

Instrument	Type	Individual/Family/Health History Factors	Community Factors	Health System Factors
CHERG Social Autopsy Tool [22]	Structured Questionnaire + Open Narrative	Mother’s age, education, literacy, marital status, age at marriage Newborn/child’s demographic factors, as well as feeding, prematurity, birth size, etc. Household possessions, husband’s education, breadwinner’s occupation Pre-pregnancy conditions, ANC provider/attendance Knowledge/recognition of and care-seeking for pregnancy, labor and delivery complications Delivery place, decision maker, factors constraining facility delivery Home delivery and newborn care Illness symptoms Infant/child care Newborn/infant/child illness recognition, care-seeking, compliance with treatment and referral advice Constraints to maternal and child healthcare seeking and referral	Place of residence, duration of continuous residence, time to reach healthcare provider Social capital (community joint action, helpful persons/groups, denial of services)	ANC content (e.g. BP, urine and blood, counseling, etc.) Delivery care (e.g. attendant, partograph use, hygiene, delivery surface) Newborn care (e.g. resuscitation, cord care, bathing, warmth, etc.) Infant/child care (e.g. vaccinations, Vitamin A) Quality of maternal and child health care and delivery services
Deshmukh et al. Social Autopsy Tool [7]	Semi-structured + Open Narrative	Care-seeking practices Treatment obtained Difficulties faced Place of death		
INDEPTH Network Social Autopsy Tool [23]	Structured Questionnaire + Open Narrative	Symptoms presented (e.g. symptoms in chronological order starting with day 1, time from first symptom to death and in relation to provider seen) Recognition of severity of symptoms Preventive behavior (e.g. bed net usage, vaccinations) Treatment-seeking behavior (e.g. type, place, and timing of treatment at home and outside the home; chronological order of providers seen, reasons for not giving care or seeking treatment, timing and sequence of providers seen, transport used for seeking care) Cost of transport/treatment/care Hospitalization of child, reason for and compliance with referral	Distance to nearest facility Availability of transportation	Care received by first and last provider visited (including waiting time and services provided) Availability of qualified doctors in the village Details of referrals
Millenium Villages Project Social Autopsy Tool [16]	Semi-structured	Sociodemographics of the mother Mother’s experiences during pregnancy Access to transportation Mother’s service utilization	Social circumstances around the death	
Njuki et al. (2014) Social Autopsy Tool [17]	Semi-structured	Sociodemographics Preventive care obtained Recognition of the signs and symptoms of illness Whether and what type of care was provided outside the home Barriers/delays encountered during care seeking Cause and place of death		Diagnostic procedures followed Type and timing of any treatment provided Quality of care provided

Methodologically, studies also varied in how deaths were identified and the length of the recall period permitted for identified deaths. Many studies (N = 6) relied upon national or large-scale mortality surveys, with a smaller number relying upon data from demographic health surveillance sites (N = 4), household surveys (N = 3) or village-based informants (N = 3). Four studies had a recall period of up to five years for each identified death, and two studies included deaths older than 5 years. One study focused on deaths within the previous year, and five studies focused on deaths within the previous six weeks. Four studies did not report the recall period.

Analytically, the studies typically used one of two frameworks to guide the analysis. Six articles used some variant of the Pathway to Survival Framework [2], six articles used the Three Delays Model [26], and 5 articles were not clear about whether they used a conceptual model to guide their analysis (see Table 2). The Pathway to Survival Framework focuses on the essential steps both inside the home and in the community to prevent child illness and includes recognition of illness, care seeking, and quality of care provided [2]. The Three Delays Model focuses on delays in deciding to seek care, delays in getting to a healthcare facility, and delays in obtaining high-quality care once at the facility [26]. Note that the definitions of delays across the six studies that focused on the Three Delays Model were not consistent (See Table 4.)

**Table 4.** Definitions of the three delays as reported in the peer-reviewed literature.

Author	Definition of First Delay	Definition of Second Delay	Definition of Third Delay
D’Ambruoso, 2010 [6]	Not defined	Not defined	Not defined
Deshmukh et al. (2016) [7]	Did not receive any home remedy and was not taken to any health facility Received home remedy and was not taken to health facility Received home remedy and was later taken to a health facility	Parents enumerated difficulties in reaching the health facility	Given no facility data, created proxies: Deaths occurring at the first health facility, or Children taken to second and subsequent facilities, or Children who were taken back from the first health facility and who died at home
Jat et al. (2015) [9]	Not precisely defined: determined by the interval between the occurrence of an obstetric complication and when the decision to seek care was made	Not precisely defined: determined by the difference of time between the decision to seek care and reaching the health care facility	Not precisely defined: determined by the time taken to initiate definitive obstetric care once the woman reached the health facility
Kallender et al. (2011) [11]	Lack of recognition of at least one severe symptom Had severe or potentially severe symptom but was treated at home Only received care at home Had severe symptom and was brought outside the home after >1 day Had only received informal care for their fatal illness as both first and last source of care Did not go for referral because of caretaker decision making	Delaying >2 hours to reach first or last provider Not going for referral because of lack of money for transport	Obtaining treatment from provider after >1 hour from first or last provider Referred because of lack of equipment of lack of medication Did not receive any treatment after visiting first or last formal provider
Upadhyay et al. (2012) [20]	Subjectively judged as a delay if the reviewers deemed that the delay seemed to have partially contributed to the death or if the delay was ‘avoidable’ by some action by either the caregiver or the health professional	Subjectively judged as a delay if the reviewers deemed that the delay seemed to have partially contributed to the death or if the delay was ‘avoidable’ by some action by either the caregiver or the health professional	Subjectively judged as a delay if the reviewers deemed that the delay seemed to have partially contributed to the death or if the delay was ‘avoidable’ by some action by either the caregiver or the health professional
Waiswa et al. (2010) [21]	Any newborn baby who died at home or where it took more than 12 hours to seek outside care	Newborn babies whose caregivers expressed problems with getting transport	Delay in receiving or failure to receive quality care at the health facility (as judged by the audit doctor)

Pursuant to the third objective of this review (to describe overarching findings across disparate populations and geographic areas), findings across the studies indicate that in general, recognition of severe symptoms for both mothers and babies is relatively high. Of the 16 manuscripts, 8 indicated high recognition of danger signs. However, subsequent action in terms of care seeking is not necessarily commensurate with the seriousness of the problem (see Table 5). Of the 8 manuscripts in which danger signs were recognized, only 3 reported respondents seeking prompt care. For example, in Uganda [11], 96% of respondents recognized severe symptoms in their children under the age of 5 before death, but a third were treated at home before going to a facility. Similarly, in Niger [12], while 95.8% of pregnant women’s caregivers reported a serious or severe symptom prior to death, 60.3% of women received no care for their illness. In Malawi, 97.8% of caretakers recognized a severe or possibly severe symptom, but only 61.1% sought or tried to seek care [14]. Table 5 illustrates that care-seeking patterns differed for neonatal, child, and maternal events. For neonates, across studies, the percentage of families who sought care for the fatal illness ranged from a low of 28.0% to a high of 76%. For children 1–59 months of age, between 78.3 and 88% of families sought care for the fatal illness. For mothers, care-seeking patterns were less reliably reported, but between 4.6 and 59.6% of mothers died at home. Across all types of events, the strongest barrier to care-seeking reported was cost, with 59.4% of respondents in one study citing cost as a primary care-seeking barrier, and 87% of respondents in another study citing cost as a reason for noncompliance with referrals.

**Table 5.** Quantitative comparisons of care-seeking patterns.

Author	Care-seeking patterns for deaths of neonates (<28 days)	Care-seeking patterns for deaths of children 1–59 months	Care-seeking patterns for deaths of mothers	Main barriers to care-seeking
D’Ambruoso et al. (2010) [6]	N/A	N/A	48% of women died at home; 52% died in facility or on way to facility Delay in deciding to seek care: 45% Delay in reaching care: 66% Delay in getting care at facility: 44%	Poor birth preparedness, unavailable/unsafe/unaffordable transport, fear of the hospital (qualitative)
Deshmukh et al. (2016) [7]	Care sought: 47.4% Delay in deciding to seek care: 62.5%	Care sought: 78.3% Delay in deciding to seek care: 42.3%	N/A	Difficulty in transit: 46%
Hildenwall et al. [8]	N/A	Care sought: 81.2% Deaths at home: 68.8%	N/A	Difficulties in illness interpretation; Financial constraints (qualitative)
Jat et al. (2015) [9]	N/A	N/A	Care sought: 100% Deaths at home: 4.6% Delay in deciding to seek care: 50%	Illness recognition; transportation problems (qualitative)
Kallender et al. (2008) [10]	N/A	Care sought: 86.0% Deaths at home: 32% Deaths en route to facility: 9%	N/A	> 1 hour walk to nearest health facility: 57%
Kallender et al. (2011) [26]	N/A	Uganda: Saw at least one provider: 80.4% Only treated at home: 19.5% Ghana: Saw at least one provider: 80.0% Only treated at home: 12.5%	N/A	Cost as reason for non-compliance with referral advice: 87% >2 hours travel time to provider: 24%
Kalter et al. (2016) [12]	Care sought: 39.7% Care sought at hospital: 6.8%	N/A	N/A	Cost: 6.0% Distance: 17.3% Transport: 18.6%
Koffi et al. (2016) [13]	N/A	Care sought: 88% Died before/en route to provider: 21.4%	N/A	Cost: 35.4% Distance: 34.5% Lack of Transportation: 30.1% Child not sick enough to warrant care: 41.7%
Koffi et al. (2015) [14]	Sought care: 61.1% Deaths at home: 36.6%	N/A	N/A	Cost: 21–74% (across groups who sought different types of care) Distance: 52–74% Lack of Transport: 49–68%
Koffi et al. (2015) [15]	Sought care: 28.0% Deaths at home: 57%	N/A	N/A	Cost:64–83% (across groups who sought different types of care) Distance:13–31% Lack of Transport:11–31%
Njuki et al. (2014) [17]	N/A	N/A	Contact with at least one provider in month preceding death: 89.9% Deaths at home: 59.6%	Cost; Distance to facility; Poor referral systems (qualitative)
Nonyane et al. (2016) [18]	Sought care: 53.2% Sought formal care first: 27.5%	N/A	N/A	Cost: 59.4% Distance: 11% Too late at night to travel: 12% Believed neonate would die anyway: 12.7% Believed traditional medicine was more appropriate: 15.8%
Tlebere et al. (2007) [19]	N/A	Many babies were seen at the facility and sent home the same day they died (qualitative)	67% of maternal deaths occurred in the hospital	Cost; No money for transportation; Belief about cause of illness; Lack of awareness of danger signs (qualitative)
Upadhyay et al. (2012) [20]	Sought care: 76% Delay in deciding to seek care: 44% Delay in reaching care (transport): 34% Delay in getting care at facility: 28%	N/A	N/A	Cost: 9.1% Care won’t benefit baby: 22.7% Delay due to belief in home treatment: 36.4% Inability to recognize danger signs: 31.2% Distance to facility: 23.5%
Waiswa et al. (2010) [21]	Sought care: 46% Delay in deciding to seek care: 50% Delay in reaching care (transport): 20.3% Delay in getting care at facility: 29.7%	N/A	N/A	Lack of recognition of danger signs: 50%

Cost, distance to facility, and lack of transportation were cited as barriers to care seeking in 14 of the 16 manuscripts. Twelve of the 16 studies provided glimpses into some of the other reasons families may not be seeking prompt attention. These included perceived low quality of care at the facility [6,9,17,21], attributing illness to spiritual and other non-medical causes [6,8,18,19], thinking that the baby would die anyway or was too sick to travel [12,14], thinking that the baby was not sick enough to seek care [14], not having a family member to accompany the woman to the facility [12,20], and needing husband’s permission to seek care [8]. Specific examples of how such barriers influence care include that in Bangladesh, 18% of mothers said they did not bring the baby for care because they thought it would die anyway [18]. In India among families experience a post-neonatal death, 104 out of 177 cases with delays at home (58.7%) reported waiting to go to the health facility to see if home remedies would to take effect [7]. In South Africa, one constraint on care-seeking commonly reported through qualitative inquiries was that the cause of the illness was attributed to witchcraft, for which western health facilities are ineffective [19].

The final objective of this review was to describe the quality of the evidence to date and to identify potential gaps in the existing literature. Table 2 shows the quality ratings of the articles, which ranged from a low of 0.67 to 1.0. Overall, the articles scored well on objective measures of quality, with the most consistent deficit being a lack of reporting on the participation rate of eligible subjects. Social autopsy research relies upon identifying and recruiting family members of individuals who have passed away, often in settings without vital registration systems or other ways to definitively determine the sampling frame. While some studies addressed this challenge by using national mortality surveys to generate a sampling frame, four of the articles included in the review did not report how many of the deaths they identified were included in their data collection efforts [8–10,21]. This limits the ability to determine potential sampling bias.

With regard to the second half of this final objective, this review identified several notable gaps in the existing social autopsy literature. The first gap is the lack of harmonization across social autopsy tools, including the domains included in the various instruments and the methodologies employed to analyze the findings. This lack of harmonization and methodological consistency makes synthesis across studies difficult. For example, some studies focused exclusively on care-seeking behaviors, while others included background sociodemographic factors such as access to toilet facilities, clean water, and other environmental factors that may impact illness exposure. Similarly, some studies reported the percentage who sought care at a facility for the fatal illness, while others reported the percentage who died at home – making it difficult to directly compare care-seeking behaviors. This is seen even among studies that relied upon the same conceptual framework, the Three Delays Model. As described, the Three Delays were operationalized differently across the different studies, making comparisons difficult. Future research designed to facilitate consistency in methodology and reporting across social autopsy research is warranted.

The second gap is – given social autopsy’s inclusion of questions about care-seeking decision-making – the lack of meaningful discussion of *the process of* decision making with regard to seeking care outside the home during critical and fatal illnesses for mothers and newborns. While some studies describe a delay in making a decision, or attribute delays to waiting for the husband to return home for the women to get permission to leave, no study addressed the potential complexity of decision making in a setting where decisions are often made as a group rather than by any one individual. Further research that attempts to clarify the cognitive, sociocultural, and communication-related processes associated with decision-making is needed.

Another gap in the social autopsy literature based upon this review are qualitative narratives that can provide deeper context to the events described through social autopsy. Of the 16 articles examined in this review, four included a qualitative component in addition to the quantitative data [6,8,17,19], and only one focused solely on qualitative results [9]. Existing social autopsy tools include an open-ended qualitative portion, yet these data are rarely included in published manuscripts. Future analyses would benefit from the explicit use of qualitative methodology to explore inconsistencies identified through the social autopsy interviews, such as why care was not sought even when danger signs were identified.

The final gap in the social autopsy literature is the absence of the explicit seeking and inclusion of perspectives of husbands and fathers surrounding these events. While invariably some of the caregivers interviewed were men, the role of husbands and fathers is not well understood. To date no published research has compared their responses to the responses of mothers or other relatives involved in the care of mothers and their babies.

## Discussion

This systematic review of the peer-reviewed literature addressing original research on maternal, neonatal, and child deaths in low-resource settings using social autopsy methods identified 16 articles representing research in 11 countries. Despite the relative nascence of the field of social autopsy research, these articles represent a diversity of geographic areas, target populations, recruitment strategies, and data collection tools. Nonetheless, there were two key findings that appeared across the studies, including: (1) high rates of recognition of serious symptoms do not always translate to high rates of care-seeking; and (2) cost, distance to facilities, and transportation are common barriers to care-seeking, yet different settings include different barriers to care-seeking, such as needing to wait for a home remedy to take effect before leaving for a western facility.

This systematic review builds upon a comprehensive review undertaken by Kalter et al. [2] that looked at articles published between 1989 and 2010 and focused on the development of social autopsy methods and the utilization of the Pathway to Survival Conceptual Framework [2]. Their review (which included 14 peer-reviewed articles pursuant to child deaths and eight related to maternal deaths) focused heavily on the elements of care-seeking that were examined in each study, as well as how the introduction of their Pathway to Survival model influenced the number of elements of care-seeking that were assessed. Since the publication of the Kalter et al. review, existing social autopsy tools have been revised and additional social autopsy tools have been developed and implemented, and thus this review moves beyond what was published in 2011. The 16 articles included in this review include three that were missed by Kalter et al. in 2011 and 11 published since 2011. Only two appear in both this review and the Kalter et al. review.

This study highlights the breadth and depth of existing social autopsy research, and followed rigorous systematic literature review methodology to ensure inclusion of all relevant articles. Starting from nearly 2000 citations, we identified 16 that included primary research using a designated social autopsy tool to explore the social and cultural factors that impact maternal, newborn, infant, or child mortality in developing country settings. Another strength of this study is that we examined not only the tools being used, but also the analytic frameworks employed. It is clear from our findings that researchers are typically choosing to use either the Pathway to Survival model or the Three Delays Model to guide their analysis, with only one published paper using both [11]. However, it is worth nothing that those articles using the Three Delays Model did not operationalize the variables in the same way, making comparison across studies difficult. While both the Pathway to Survival model and the Three Delays Model have yielded important findings, the reliance on these models above all others suggests that the research literature might benefit from the development and testing of alternative frameworks.

Despite the strengths, this review was limited to English-language articles published in indexed journals, and it is possible that research conducted in non-English-speaking countries may not be adequately represented. This review is also limited by the lack of detail included in many published manuscripts regarding the content, duration, domains, and individual items included in their social autopsy tools. It is also difficult to determine how the instruments have been revised over time and/or how they have been adapted to different contexts.

In sum, this review suggests a need for harmonized social autopsy tools and methodologies, as well as a need to better understand the complexities of decision making around care seeking for serious illnesses among mothers and babies. Given the study findings that care-seeking is more common for older children than for newborns, it also highlights the need for deeper inquiry using qualitative methodology to unpack inconsistencies in care seeking, even in the face of recognition of the severity of symptoms. The studies included in this review indicate that cost, distance, and transportation – while common barriers to care seeking – are not the only barriers for women and families experiencing life-threatening complications. Until such barriers are understood and addressed through locally appropriate means, delayed or lack of care-seeking will continue to serve as a barrier to improving maternal, neonatal, infant, and child outcomes. This study also illustrates the importance of including, articulating, and expanding upon the potentially unique perspectives of men in influencing care-seeking for mothers and their children.

## Paper context

This paper builds on a previous comprehensive review of the literature to illustrate how far the field of social autopsy has come since 2005. We identified 17 articles using five different social autopsy tools, with the Pathway to Survival and Three Delays models most commonly used for analysis frameworks. Across studies, recognition of danger signs was high, while subsequent care seeking was not. Barriers to care seeking were reported that varied by location.
